# Prospective Early Clinical, Radiological, and Kinematic Pedobarographic Analysis Following Subtalar Arthroereises for Paediatric Pes Planovalgus

**DOI:** 10.7759/cureus.6309

**Published:** 2019-12-06

**Authors:** Yvonne-Mary Papamerkouriou, Rohan Rajan, Samena Chaudhry, Preetham Kodumuri, Helen Evans, Martin Kerr

**Affiliations:** 1 Orthopaedics, Panagiotis and Aglaia Kyriakou Children's Hospital, Athens, GRC; 2 Orthopaedics, Royal Derby Hospital, Derby, GBR; 3 Physiotherapy, Royal Derby Hospital, Derby, GBR

**Keywords:** pes planovalgus, arthroereisis, pedobarograph

## Abstract

Introduction

Arthroereises implants mechanically block eversion and limit subtalar motion. They are used in children with pes planovalgus in order to correct the valgus deformity. In this study, we aimed to objectively assess children with flatfoot before and after the insertion of the Kalix II implant, clinically, radiologically and by kinematic pedobarographic analysis.

Materials and methods

Six children (12 feet) were treated by the insertion of the Kalix II implant (Integra LifeSciences, Plainsboro, NJ). Patients completed the Manchester Oxford Foot Questionnaire (MOXFQ) preoperatively and at six months post operatively. Radiological outcome was assessed by lateral (L) and anterior posterior (AP) foot weight-bearing radiographs taken pre operatively and post operatively. Pedobarographic data was obtained pre operatively and at six months post operatively using a 1 meter RS Scan Footscan (RSscan International, Olen, Belgium) pedobarograph. In addition, patients underwent gait analysis pre and post operatively.

Results

Mean age was 11.05 +/-3.24 years (range 6.2 to 15.5 years). In all cases, screw removal was carried out at between 15 to 18 months post insertion. The mean pre op MOXFQ score was 55.3 +/-9.68 which reduced to 34.3 +/-15.66 post operatively with a p value < 0.00001 which was statistically significant. Mean Meary's angle preop was -15.21+/-5.51 degrees which corrected to -7.57+/-4.62 post op with a p value=0.00001. The mean calcaneal pitch before surgery was 11.96+/-3.8 which increased to 14.98+/-3.85 with a p value =0.00067. The first MTH: fifth MTH peak pressure ratio pre operatively was 4.53+/-2.78 which was found to reduce significantly post operatively to 1.35+/-0.97 (p=0.04), indicating a lateral shift of the foot pressures.

Conclusion

There were statistically significant improvements in the patient-reported MOXFQ, radiological improvements, and pedobarographic changes, indicating a lateral shift of the foot pressures. There were no complications.

## Introduction

Flatfoot is a common paediatric problem. In the vast majority of cases, these are physiological in nature and improve spontaneously as the child grows. A differentiation between pes planus - flatfoot and pes planovalgus - flat foot with valgus of the calcaneus on weight bearing needs to be made. Clinical evaluation is made to differentiate between a rigid and a flexible flat foot.

In stance, the heel is found to be in valgus, which corrects into neutral or varus on forefoot weight bearing with restoration of the medial longitudinal arch, in flexible pes planovalgus. Children with this deformity usually present with medial midfoot weight bearing, mild genu valgum and parental concern. Tarsal coalition is the usual cause for a rigid pes plano valgus, where this correction does not occur. These patients usually present with a stiff painful flat foot. Consideration should also be given towards Achilles and gastrocnemius contractures (differentiated by the Silfverskiold test) associated with this condition. As the calcaneus is in valgus, the Achilles tendon is found to be contracted [1].

The subtalar joint comprises the talus, calcaneus and navicular and the intervening interosseous talocalcaneal and calcaneonavicular ligaments (spring ligament) and multiple joint capsules. These function as a unit. Flatfoot should be thought of as a three-dimensional deformity where the hindfoot is pronated, the subtalar joint is dorsiflexed and externally rotated, the midfoot abducted and forefoot supinated [2].

Initially, conservative treatment is advocated such as physiotherapy, reassurance, medial arch supports or UCBL orthotics. However, there is no evidence supporting the use of orthotics to correct this deformity [3]. It has been suggested that orthotics could, in fact, lead to dependency and long term negative psychological effects [3-5].

When these have failed, in patients with continued pain and dysfunction, surgical intervention is offered. Arthroereisis procedures were introduced between 1946 and 1977 to restrict excessive subtalar joint eversion by placing a bony block in the sinus tarsi [6-9]. Bone grafts were found to resorb over time leading to recurrence. Arthroereisis with synthetic implants was introduced in the late 1970s as these do not resorb, allowing for a greater time for bone remodeling [2]. Subotnick first described using a block of silicone elastomer in the sinus tarsi [10]. Although considered extra articular, if inserted appropriately, it mechanically blocks eversion and blocks subtalar joint motion with an intra articular effect [11-12].

During pronation, the sinus tarsi closes as the lateral process of the talus glides anteriorly towards the prethalamic surface of the calcaneus. During foot supination, the sinus tarsi opens. An arthroereisis implant limits this motion, limiting hindfoot pronation. This prevents the lateral process of the talus from advancing towards the floor of the sinus tarsi, causing the subtalar joint to be inverted. It is postulated that extra articular arthroereises implants temporarily correct the valgus deformity in children allowing for bone remodeling to lead to a permanent correction following its removal [13].

Sixty percent of the talar surface is covered by articular cartilage which moves simultaneously with the peritalar structures [13]. The subtalar joint is a single axis joint acting as a hinge connecting the talus and calcaneus. When the subtalar joint axis is inclined 45 degrees from the transverse plane, rotation of the vertical part of the joint is coupled to equal rotation of the horizontal part of the joint [14]. A more horizontally aligned axis such as in planovalgus causes a greater rotation of the horizontal part for a given rotation of the vertical part. This allows greater supination/pronation at the longitudinal axis of the horizontal segment for a given external/internal rotation of the vertical segment.

Vogler classified the biomechanical mode of action of subtalar arthroereisis implants into three distinct modes: “axis altering”, “impact blocking” and “self locking” [15]. This last mechanism of action is that which is employed by the implant in our study as it is inserted into the main axis of the sinus tarsi, supporting the talar neck, thus avoiding contact between the lateral process of the talus and the sinus tarsi floor, limiting talar adduction and plantar flexion.

In this study, we aimed to objectively assess children with flatfoot before and after treatment by insertion of the Kalix II (Integra LifeSciences, Plainsboro, NJ) arthroereisis implant, clinically, radiologically and by kinematic pedobarographic analysis.

## Materials and methods

Six children (12 feet) with symptomatic painful correctible flexible pes planovalgus were included in our prospective study where the Kalix II subtalar arthroereisis implant consisting of a tapared tintanium alloy body and ultra-high molecular weight polyethelene shell was employed. All patients had a failed trial of conservative treatment including orthoses and physiotherapy. None of our patients had tarsal coalition. All our patients were assessed especially not to require any adjunctive soft tissue procedures in addition to the insertion of the subtalar arthroereisis implant. This was important in order to allow us to assess specifically the changes afforded by the implant alone, post operatively.

All patients were given a general anaesthetic and were positioned supine. Image intensifier was used in theatres. A 1-cm incision was performed just anterior and plantar to the tip of the lateral malleolus, allowing for blunt dissection down to the sinus tarsi. The Kalix II Viladot lever was inserted from the anterior to posterior direction into the sinus tarsi to elevate the talus whilst the forefoot was pronated to restore the medial longitudinal arch. Trial implants of increasing size were inserted after the removal of the Viladot lever until a stable trial was found. The appropriate implant was then inserted with the lateral border of the implant being flush with the lateral border of the talus as confirmed by image intensifier. Surgical incision was closed in layers with subcuticular closure to the skin. A below-knee weight-bearing plaster cast was applied for the first four weeks post operatively. This was mainly to rest the soft tissues and avoid early extrusion, as well as to assist in pain relief.

All patients were asked to complete the validated patient-reported Manchester Oxford Foot Questionnaire (MOXFQ) preoperatively and at six months post operatively. MOXFQ is a 16-item patient-reported outcome (PRO) measure developed and validated for use in studies assessing outcome following foot and/or ankle corrective surgery. Scores for each domain are calculated by summing the responses to each item within a given domain. Raw scores can be converted to a 0-100 metric where 100=most severe.

Radiological outcome was assessed by lateral (L) and anterior posterior (AP) foot weight bearing radiographs taken pre operatively and post operatively. In each case, the Meary's angle (talus - first metatarsal angle) and calcaneal pitch (angle formed by line passing from plantar most surface of calcaneus to inferior border of calcaneocuboid joint and line from plantar surface of calcaneus to plantar surface of fifth metatarsal head) of the lateral weight-bearing radiographs were calculated.

Children underwent gait analysis pre and post operatively. Pedobarographic data was obtained pre operatively and at 6 months post implant insertion at our gait laboratory using a 1 meter RS Scan Footscan (RSscan International, Olen, Belgium) pedobarograph, capturing data at 200 Hz. A minimum of four passes were made to capture at least six clear footprints on each side (right and left).

Parametric data was expressed as mean+/- standard deviation (SD). The SPSS 17.0 software (SPSS, Chicago, IL, USA) was employed for statistical analysis. Paired Student’s t-test was used for comparisons for pre operative and post-operative results. A p value of <0.05 was considered statistically significant.

## Results

All six children in our prospective study underwent bilateral procedures. Mean age 11.05 +/-3.24 years (range 6.2 to 15.5 years). In all cases, screw removal was carried out between 15 to 18 months post insertion. There were no complications in our cohort of patients.

MOXFQ scores

The mean pre-op MOXFQ score was 55.3 +/-9.68 which reduced to 34.3 +/-15.66 post op with a p value < 0.00001 which was found to be a statistically significant result. In the MOXFQ the maximum score is 100 which corresponds to the most severe result.

Radiographic correction

Mean Meary's angle preop was -15.21°+/-5.51° degrees which corrected to -7.57°+/-4.62° post-op with a p value=0.00001. In a normal foot, Meary’s angle is 0°. The negative value indicates the convex of the angle is downward, as is expected in flatfoot. In our study, whereas Meary’s angle did not correct to normal, it reduced, indicating improvement. The mean calcaneal pitch before surgery was 11.96°+/-3.8° which increased to 14.98°+/-3.85° post op with a p value =0.00067. Angles between 10° and 20° are indicative of pes planus. In our study, the angle increased, indicating improvement even though, it remained below the normal range.

Pedobarographic data

We analysed the peak pressures under the first and fifth metatarsal heads (MTH) pre operatively and post operatively in kilopascals (kPa). Before surgery, the mean first MTH peak pressure was 218.42+/-109.56 which reduced postoperatively to 113.82+/-79.71 (p=0.0016). The mean preoperative fifth MTH peak pressure was 65.08+/-32.83 increasing to 88.58+/-59.17 (p=0.281). The first MTH: fifth MTH peak pressure ratio pre operatively was 4.53+/-2.78 which was found to reduce significantly post operatively to 1.35+/-0.97 (p=0.04), indicating a lateral shift of the foot pressures (Table 1).

**Table 1:** Results Pre- and post-operative MOXFQ, radiological, and kinematic data. MOXFQ: Manchester Oxford Foot Questionnaire; MTH: metatarsal heads.

	Pre op (+/-SD)	Post op (+/-SD)	P value
MOXFQ	55.3 (9.68)	34.3 (15.66)	0.00001
Meary angle	-15.21 (5.51)	-7.57 (4.62)	0.00001
Calcaneal pitch	11.96 (3.8)	14.98 (3.85)	0.00067
1^st^ MTH peak pressure (kPa)	218.42 (109.56)	113.82 (79.71)	0.0016
5^th^ MTH peak pressure (kPa)	65.08 (32.83)	88.58 (59.17)	0.281
1^st^:5^th^ MTH PP ratio	4.53 (2.78)	1.35 (0.97)	0.04

**Figure 1:**
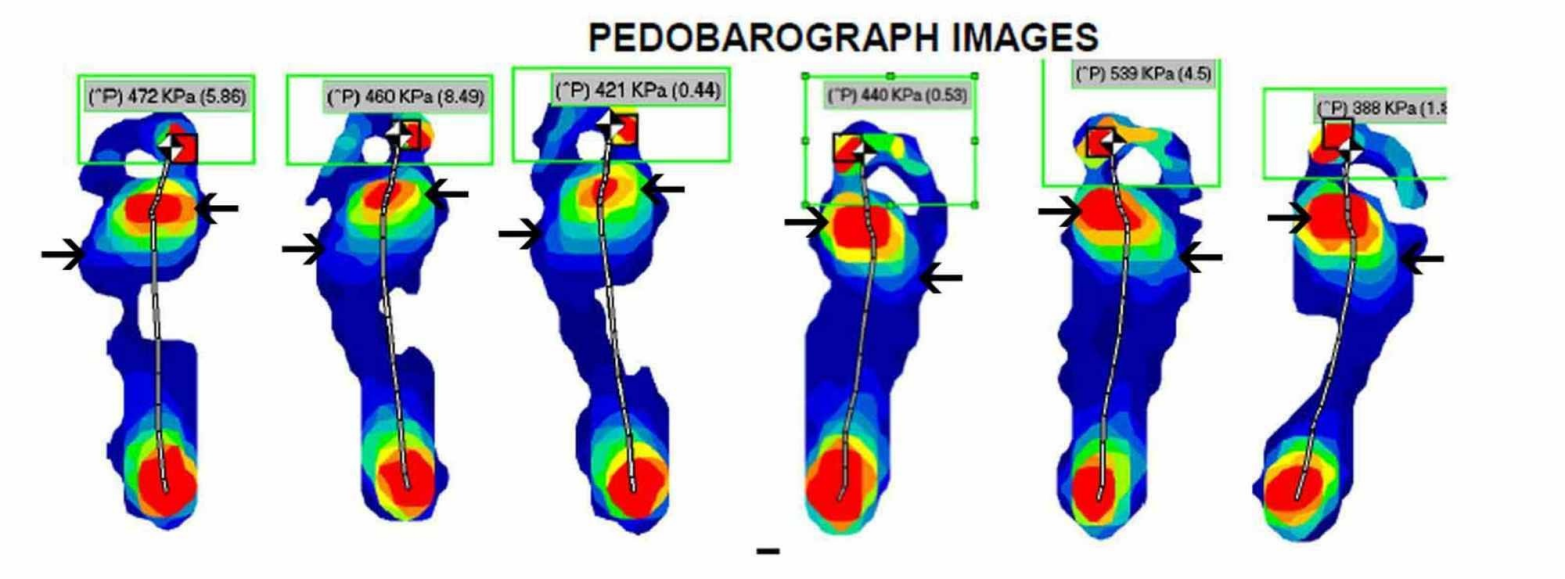
Pre-operative pedobarograph pressure images Pre-operative pedobarograph pressure images of one of the patients in our cohort. There is an increase in pedobarographic pressures under the first metatarsal head (MTT) and a decrease under the fifth MTT.

**Figure 2:**
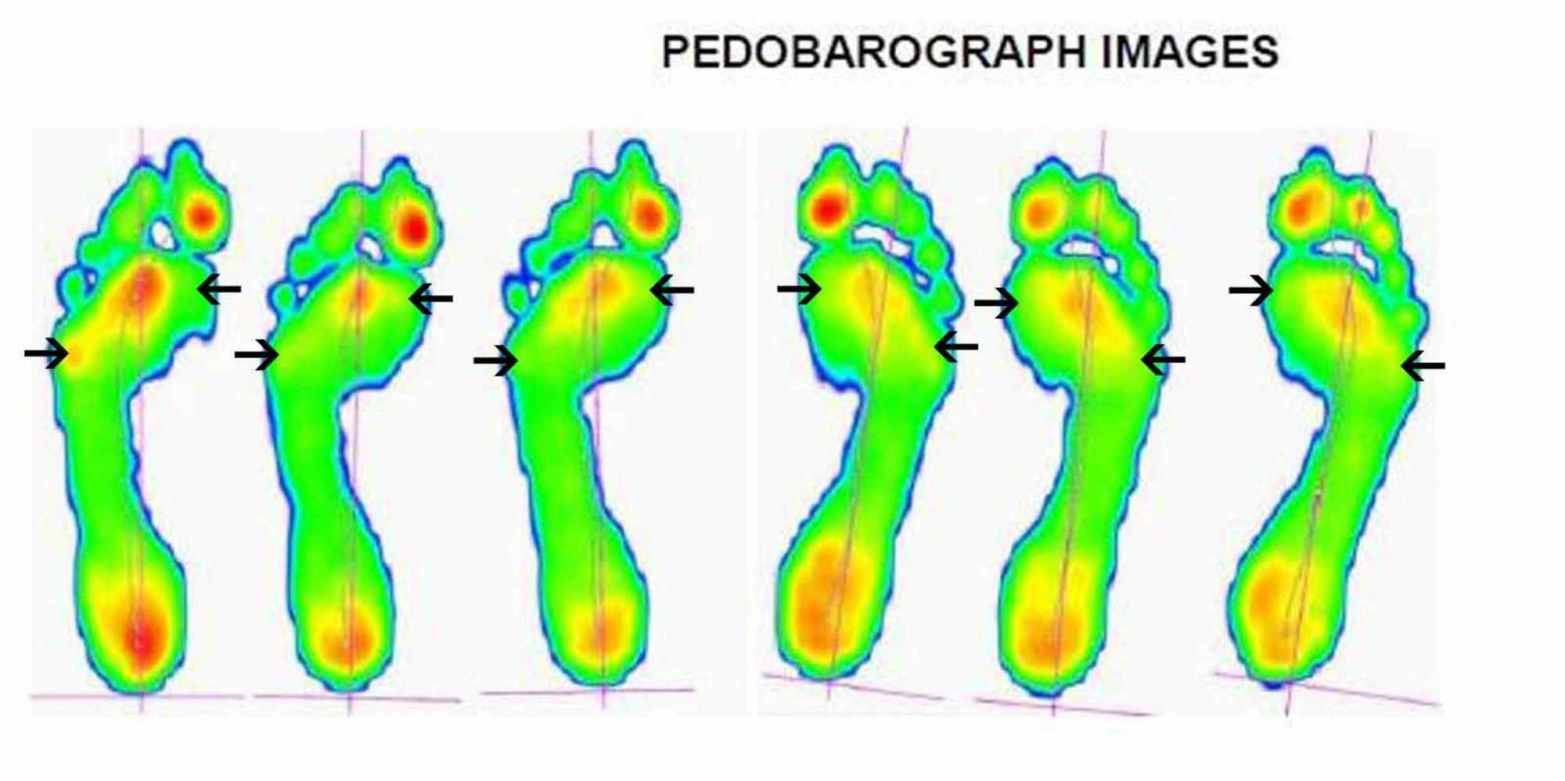
Post-operative pedobarograph pressure images Post-operative pedobarograph pressure images of the same patient. There is a decrease in pressures under the first metatarsal head (MTT) and an increase under the fifth MTT head indicating a lateral shift of pressures.

We allowed for post operative swelling to settle before performing the above measurement at around the six-month post operative period. We postulated that before corrective surgery, there is an increase in pedobarographic pressures on the medial side of the foot as it is pronated in pes planovalgus. These should be reduced as the foot is corrected into some supination, allowing for a reduction in medial pressures and an increase in lateral pressures of the foot. This is apparent in the pedobarographic images of one of the patients in our cohort, before and after the procedure (Figures 1-2). Our study has demonstrated a statistically significant reduction in the peak first metatarsal head pressure post operatively while the increase in the peak fifth metatarsal head pressure post operatively was not statistically significant. However, when we analysed the ratio of the first to fifth metatarsal peak pressures, which we feel is a more meaningful measure of the redistribution of the foot pressures than the raw individual metatarsal head pressures, we found a statistically significant redistribution of foot pressures towards the lateral side of the foot and away from the medial side of the foot.

**Figure 3:**
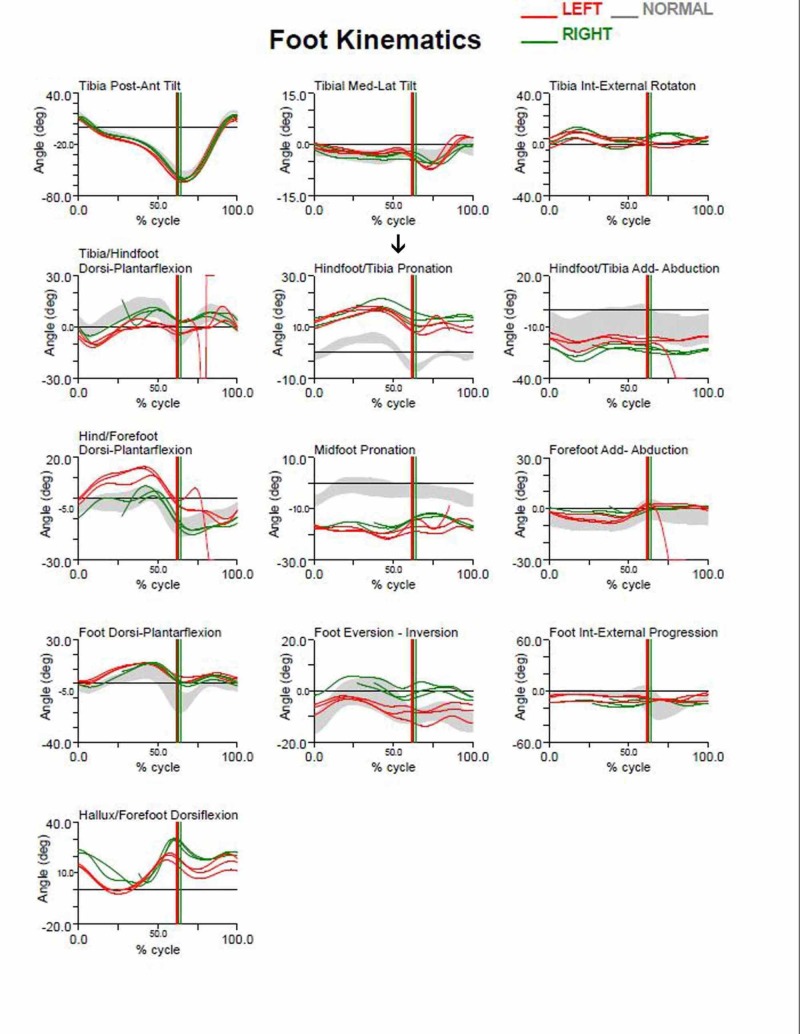
Pre-operative gait analysis graphs Pre-operative gait analysis graphs of the aforementioned patient indicating increased hindfoot pronation.

**Figure 4:**
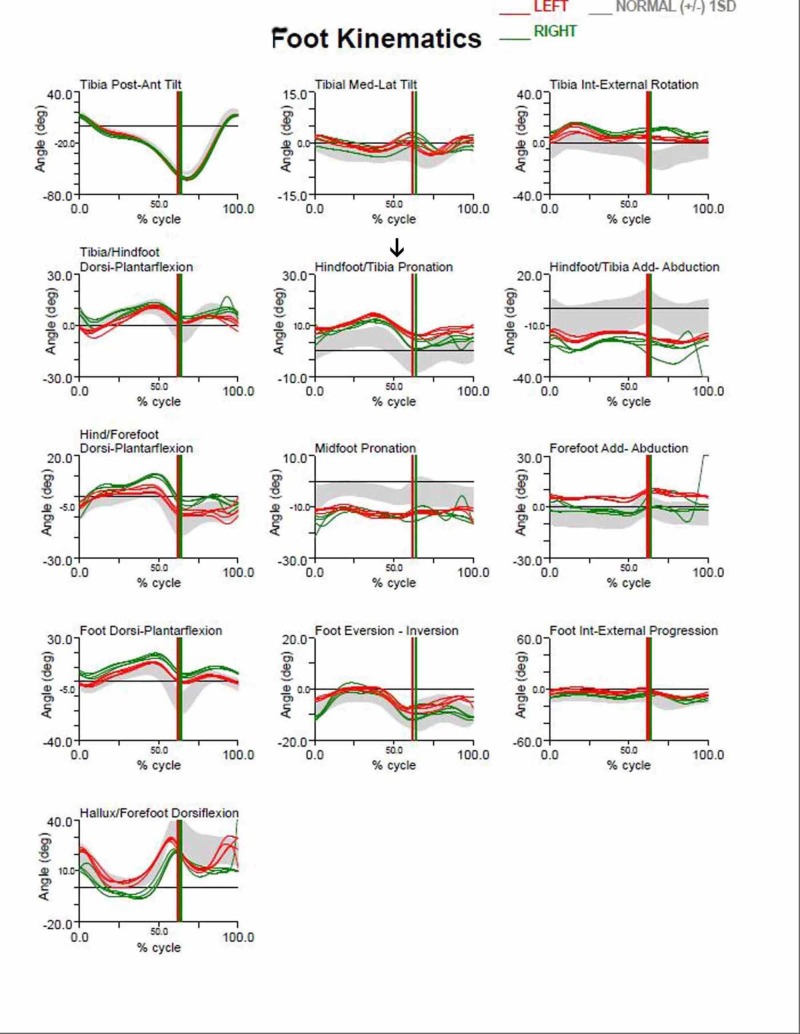
Post-operative gait analysis graphs Post-operative gait analysis graphs of the aforementioned patient indicating a decrease in hindfoot pronation.

**Figure 5:**
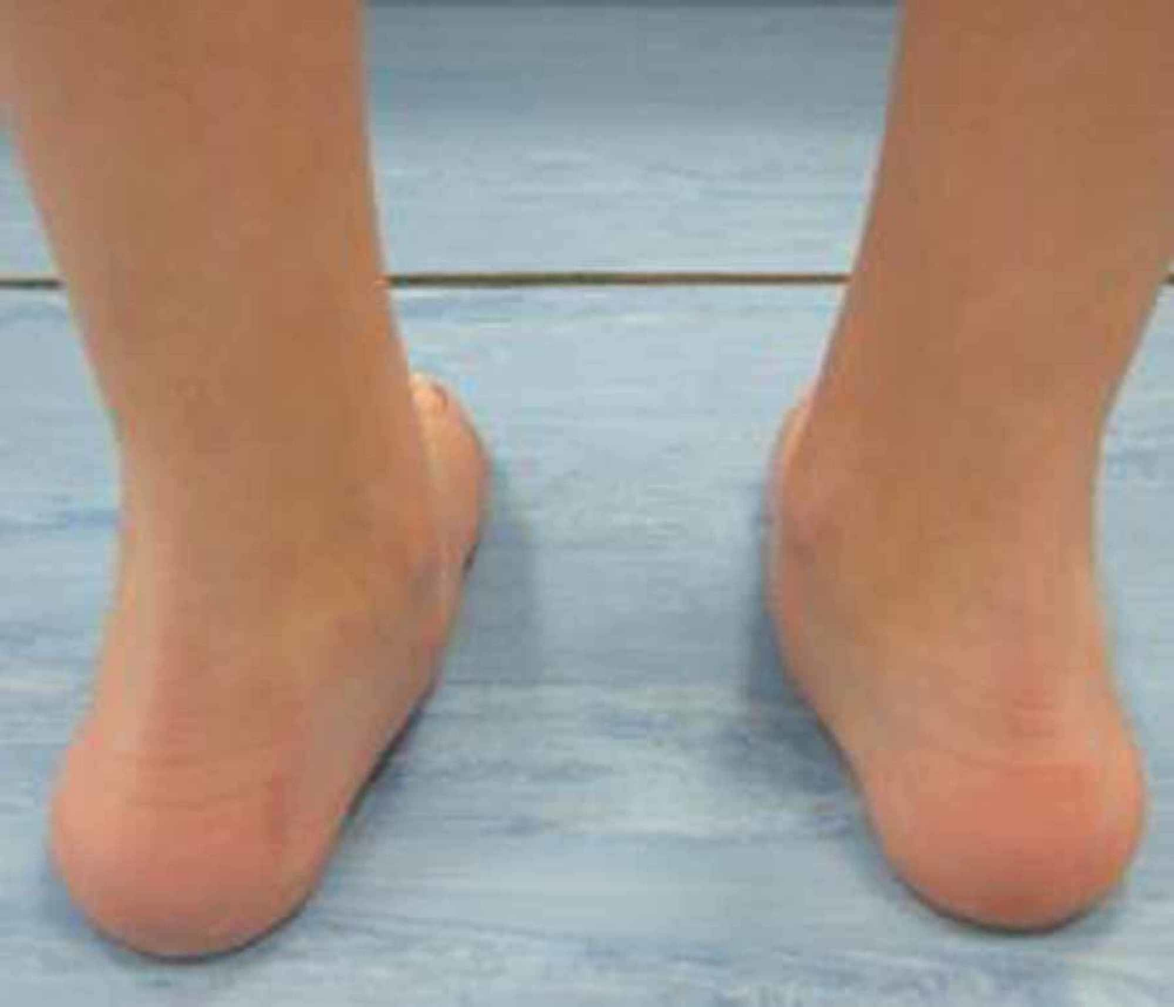
Pre-operative gait analysis photo of heel valgus Pre-operative gait analysis photo of the same patient indicating prominent heel valgus.

**Figure 6:**
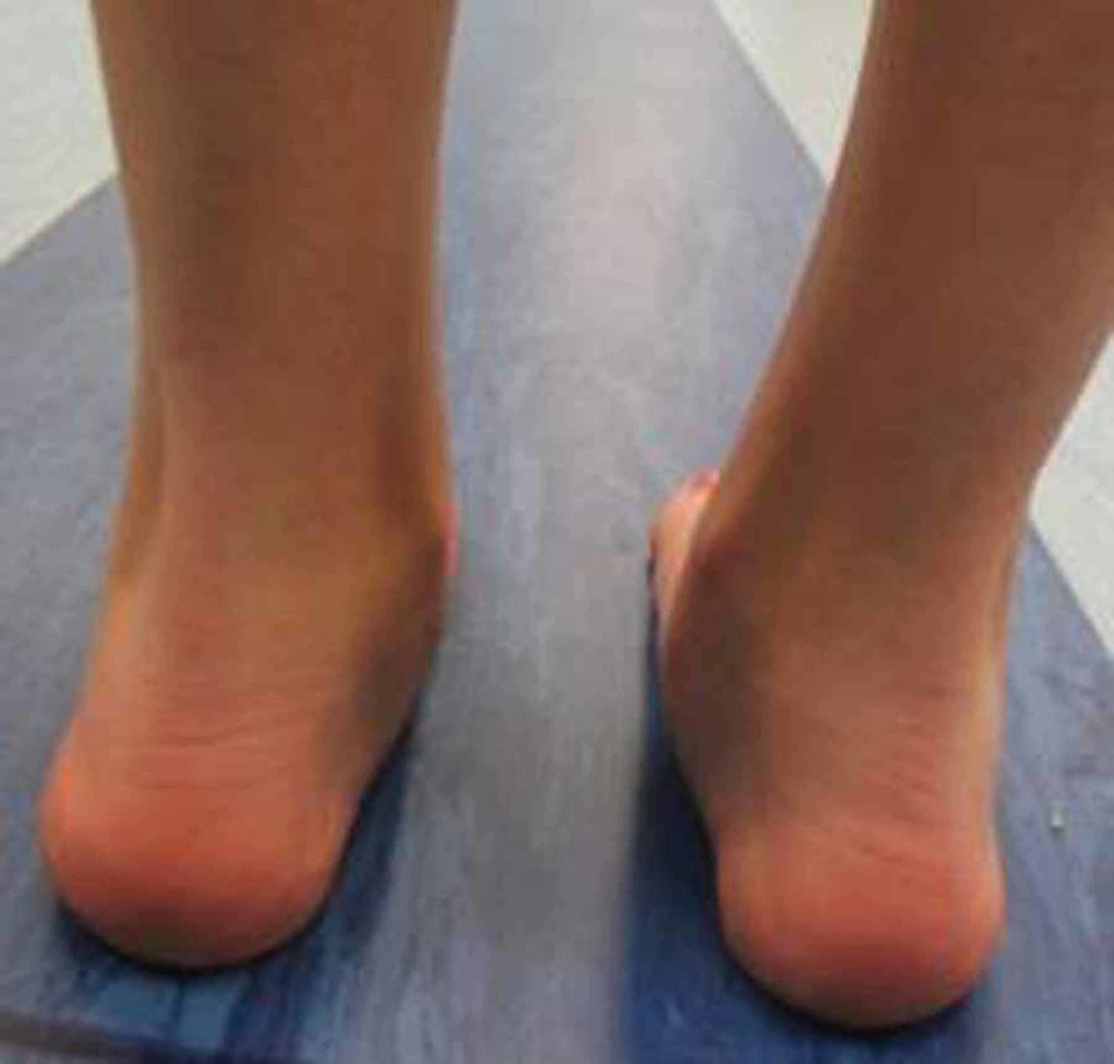
Post-operative gait analysis photo of heel valgus Post-operative gait analysis photo of the same patient indicating a decrease in heel valgus.

**Figure 7:**
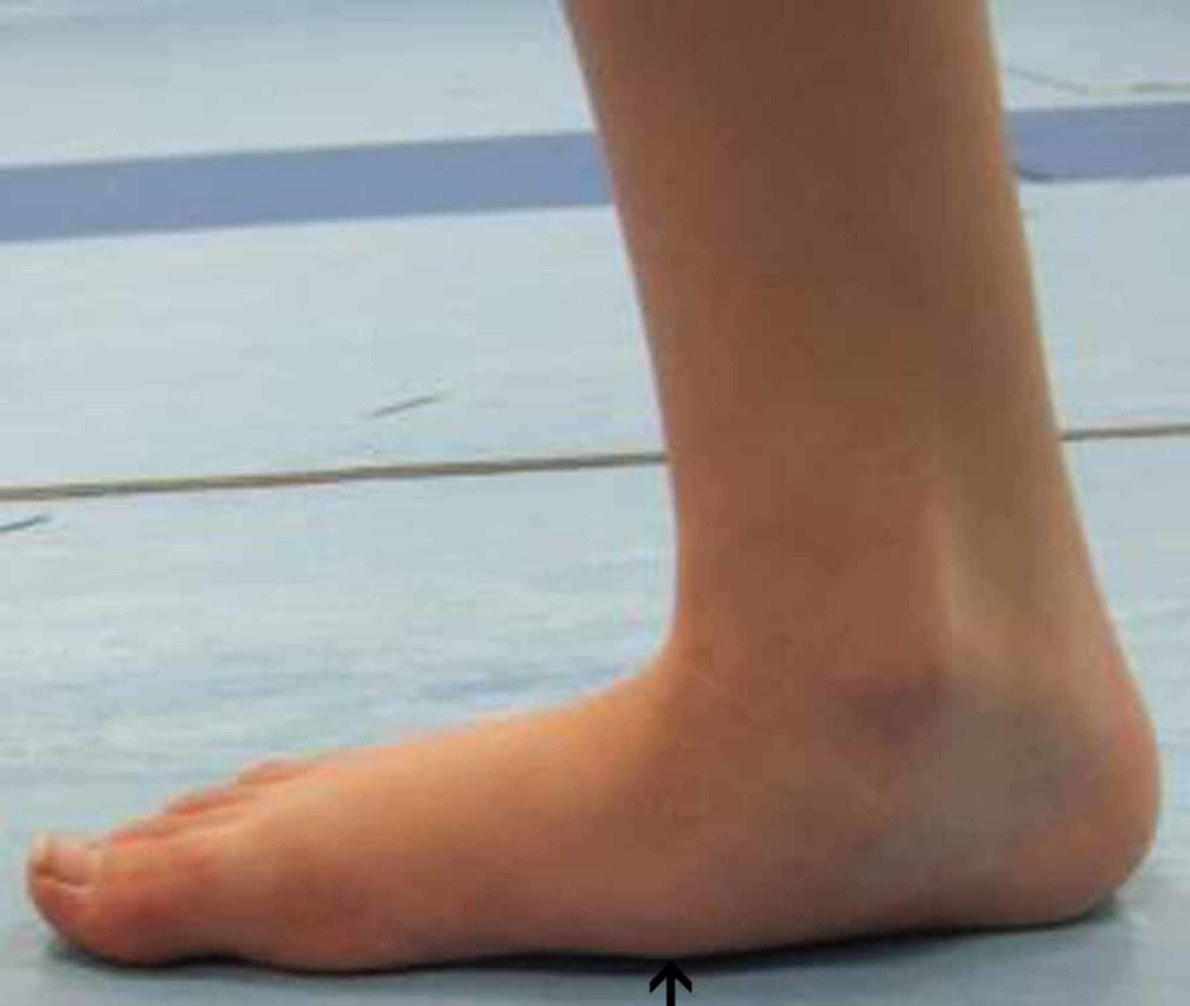
Right foot pre-operative gait analysis photo of the medial arch Right foot gait analysis photo of the medial arch of the same patient where there is evident flatening of the arch.

**Figure 8:**
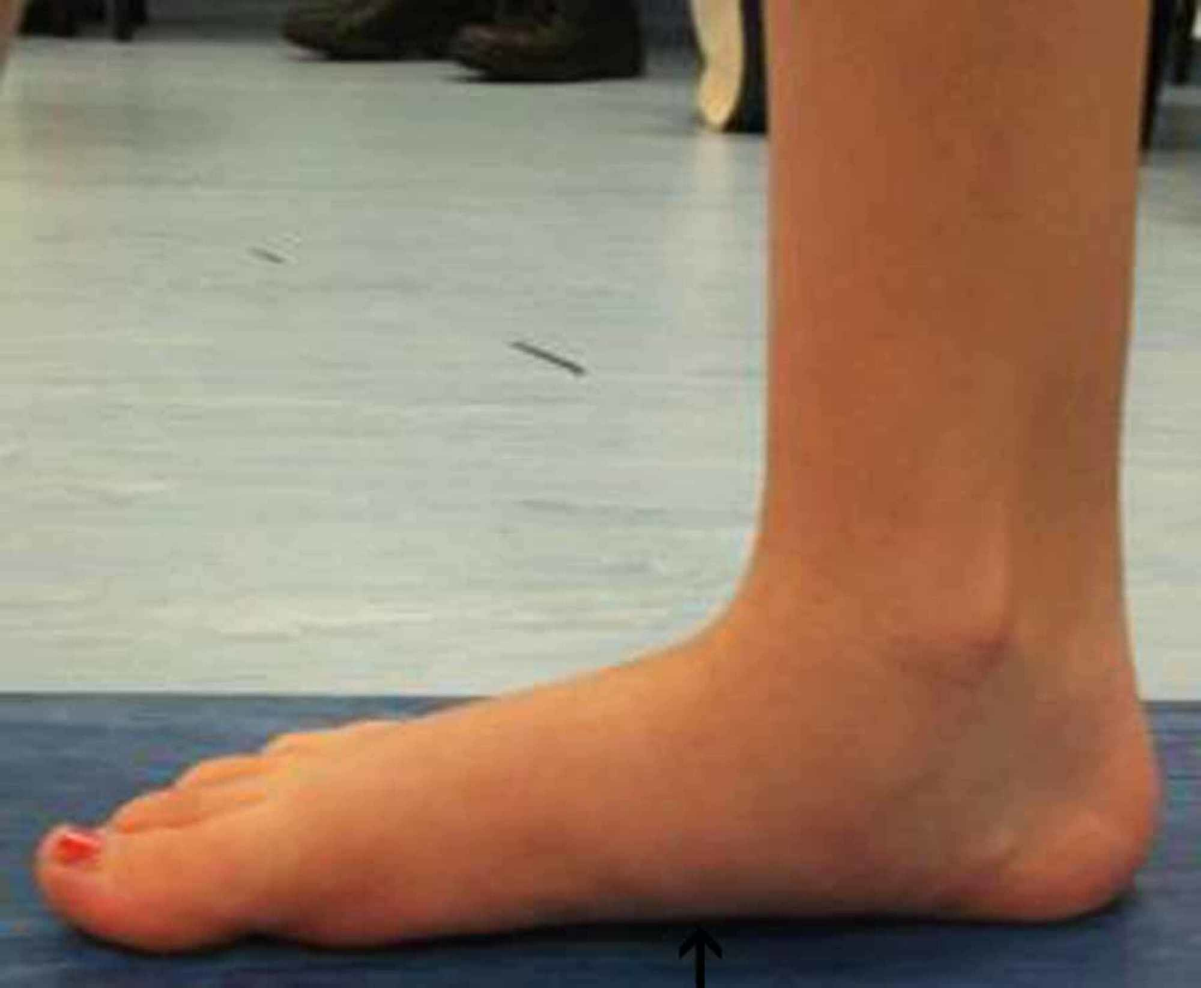
Right foot post-operative gait analysis photo of the medial arch Right foot post-operative gait analysis photo of the medial arch indicating elevation of the arch.

**Figure 9:**
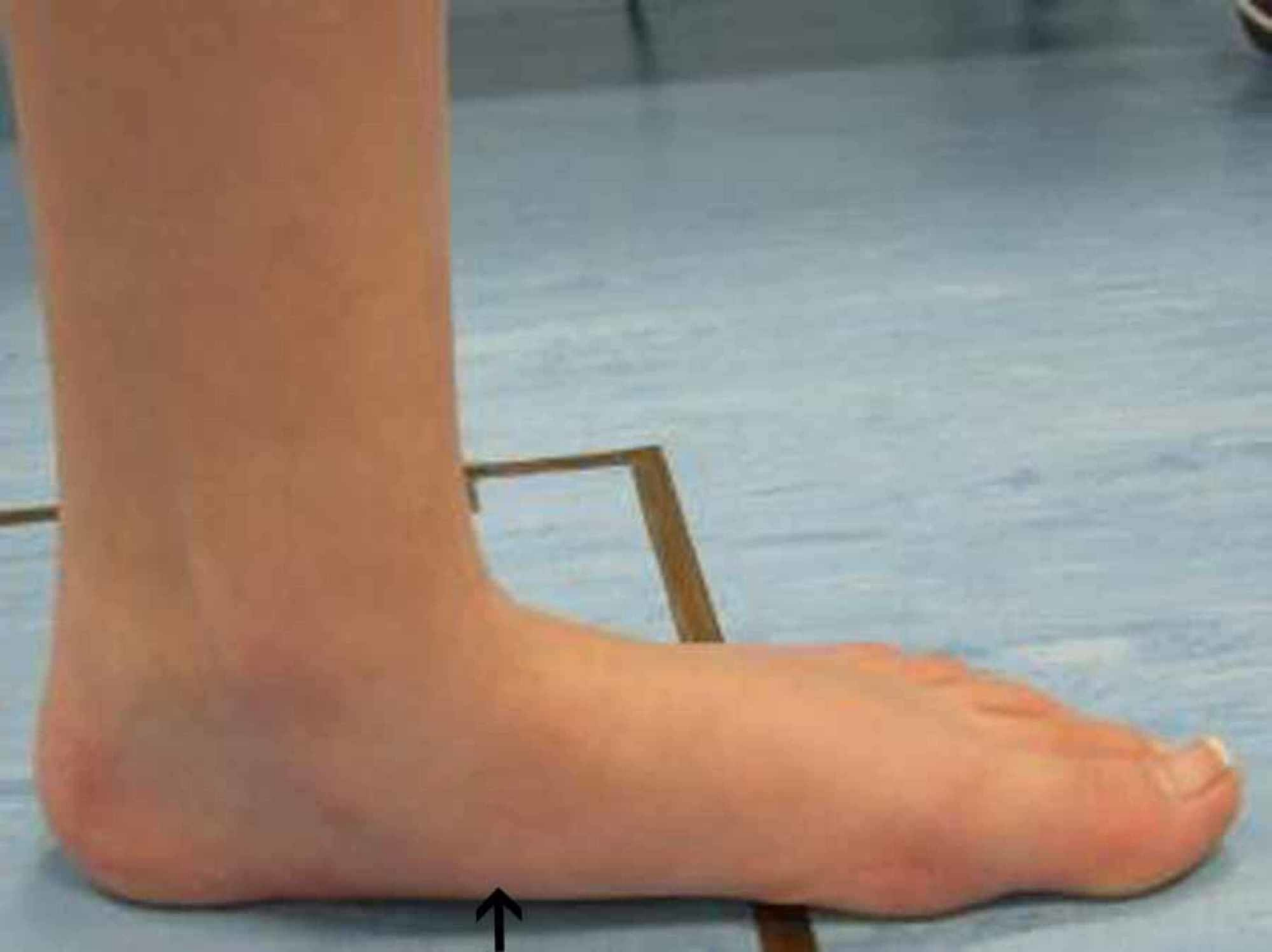
Left foot pre-operative gait analysis photo of the medial arch Left foot pre-operative gait analysis photo of the same patient indicating flattening of the medial arch.

**Figure 10:**
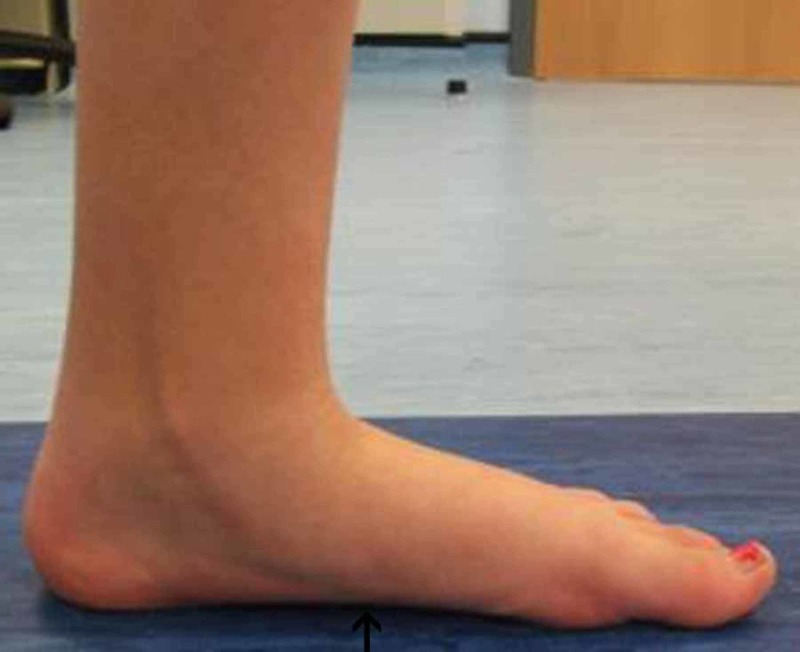
Left foot post-operative gait analysis photo of the medial arch Left foot post-operative gait analysis photo of the medial arch indicating elevation of the arch.

In addition, we observed the limiting of hindfoot pronation, as demonstrated by the pre- and post-surgery gait analysis graphs of the aforementioned patient (Figures 3-4). Gait analysis photographs indicate a reduction in heel valgus (as seen in Figures 5-6) as well as an improvement in the medial arch post operatively (Figures 7-10) in the same patient.

**Figure 11:**
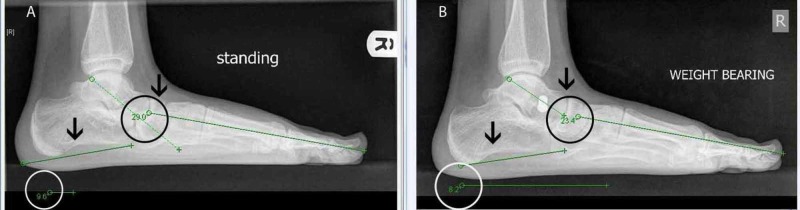
X-rays of the right foot Meary's angle and calcaneal pitch A) Right foot pre-operative radiological angles, specifically Meary's angle and calcaneal pitch in a lateral standing X-ray of the foot. B) Right foot post-operative radiological angles. There is a decrease in Meary's angle indicating improvement; however, there is a slight worsening in the calcaneal pitch, as indicated by its decrease.

**Figure 12:**
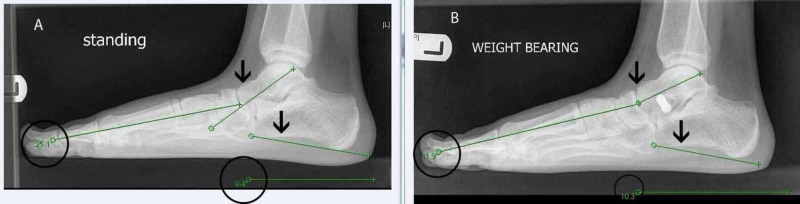
X-rays of the left foot Meary's angle and calcaneal pitch A) Left foot pre-operative radiological angles, namely Meary's angle and calcaneal pitch, in a lateral standing X-rays of the foot. B) Left foot post-operative radiological angles. There is an improvement in both angles, indicated by the decrease in Meary's angle and increase of calcaneal pitch.

Furthermore, our prospective study has demonstrated an improvement in the radiological calcaneal pitch and Meary’s angle measured at six months post surgery. With respect to radiological angles, although in specific cases the post operative angles revealed some under correction, the improvement post operatively was statistically significant overall. We have included X-rays of the already stated patient where there was an improvement in Meary’s angle post operatively in both feet. Calcaneal pitch improved slightly in the left foot but worsened in the right (Figures 11-12). 

## Discussion

Arthroereisis for the treatment of severe pes planovalgus has been used for over 50 years. The use of implant materials and shapes vary but no study has demonstrated an advantage of one over another [16]. Vedantam et al. reported satisfactory results in 96% of feet in a study of 78 children with neuromuscular flexible flatfeet where 140 arthroereisis procedures were performed utilizing STA-peg implants. Assessment was based on radiological angle improvement as well as reduction of hindfoot valgus and pain [17]. Giannini et al. reported 4-year results of subtalar arthroereisis for 21 children with bilateral flexible flatfeet using a bioresorbable implant, finding improvement in clinical results, radiological angles and footprint grades [18]. Fernandez de Retana et al. found that the Viladot foot prints and radiographic angles improved post operatively, in a study of 97 feet where the Kalix implant was employed [19]. The Viladot 4 category footprint classification used is a simple visual model which does not allow quantifying of pre and post operative differences. Many other publications for subtalar arthroereisis include Achilles tendon lengthening [12,20-23]. Overall, these studies have demonstrated an increase in dorsiflexion, decreased foot pain, improvement of radiographic angles and improvement in foot print following this procedure [18,21,24-25]. De Pellegrin et al. found that following removal of the implant there was the maintenance of the correction in an evaluation of 76 patients (121 feet) who were followed up after screw removal, which occurred on average 2.9 years after SESA (subtalar extra-articular screw arthroereisis). The evaluation was based on radiological angles [26].

Cao et al. compared radiographic results as well as pain and function pre and post application of the Kalix II arthroereisis system in 27 feet. The study concluded that the application of the Kalix II system combined with dissection of accessory navicular and reconstruction of the tibialis posterior tendon is an effective therapy for juvenile flexible flatfoot [27].

Bernasconi et al., in their review of the role of arthroereisis of the subtalar joint for flatfoot, have concluded that only level IV and V evidence is available. There is only one level II comparative non-randomised study, which does not provide strong recommendation. It was suggested that a validated patient outcome measure needed to be used as many of the studies still used non-validated scores. No firm recommendations were made about the timing for the removal of the implants or even not removing them [28]. Older studies recommended leaving the implant in situ for up to two years before removal allowing for bone and soft tissue remodelling [10,8]. More recent literature recommends removal between 6-18 months [29-30].

A recent literature review reported concerns about a high complication rate in 4%-18% of cases [24]. Reported complications include malposition of the implant, improper correction of the deformity, extrusion of the implant from the sinus tarsi, foreign body reaction to the implant, peroneal spasm and persistent foot pain. These complications are usually treated by implant removal. We removed the implants at around the 18 month period, following literature recommendations, as we were concerned about any polyethelene debris.

To date, we found no other study in the existing literature evaluating the outcomes of arthroereisis by pedobarographic evaluation. Our study was primarily concerned with the objective changes or improvement in the pedobarographic pressures following the insertion of the subtalar arthroereisis implant.

Limitations of this study include the small numbers treated, although they provided adequate statistical analysis, as well as the lack of a control group of normal-arched children. We continue to follow up with these patients until skeletal maturity and post removal of the implant. We hope to be able to report on their pedobarographic changes at one and two years post removal of the implant with a larger series of patients. However, we felt it important to highlight the improvements in pedobarographic, radiological and MOXFQ outcome in the early stages pre removal of the implant.

## Conclusions

In our small prospective series, there were no complications. We have been impressed particularly by the objective statistically significant improvements in the patient-reported MOXFQ, radiological improvements, and pedobarographic changes. We feel that the arthroereisis procedure is safe and simple if performed correctly, with attention to the correct placement of the implant in carefully selected, appropriate patients. We intend to follow up on this study over a longer period.
